# Size matters: modeling the effects of body shape on locomotive behavior in the nematode *C. elegans*

**DOI:** 10.1186/1471-2202-13-S1-P163

**Published:** 2012-07-16

**Authors:** David R Williamson, Netta Cohen

**Affiliations:** 1 School of Computing, University of Leeds, Leeds, West Yorkshire, LS2 9JT, UK

## 

The microscopic nematode worm, *C. elegans*, has a hard wired and comprehensively mapped neural circuit, with only 302 neurons in the adult. The organism's simplicity and the wealth of information available about it make *C. elegans* an inviting target for computer modeling. Locomotion is the clearest measure of the worm's behavior, and the undulatory waves used in the worm's movement are directly affected by the shape of the animal's body. As the worm develops from a larva to adulthood its body grows and changes in shape. More dramatically, mutant *C. elegans* exist with bodies that are considerably longer or shorter than in the wildtype, and these worms have severe locomotion phenotypes.

Previous models for locomotion have often concentrated on either the worm's nervous system or body, modeling only one area in detail and greatly simplifying the other. This neglects important interactions between the physical and neural aspects of the worm's behavior, such as touch response, proprioception and the effects of its surroundings. We use an integrated neuromechanical model that incorporates the worm's neural circuit for locomotion, its body and its environment. We have previously used the model to predict that the L1 larva maintains robust forward locomotion using a similar gait to the adult worm, and now examine the behavior of the body shape mutants dpy-1, dpy-11 and lon-2, both in liquid and on gel. Testing model predictions against data from biological experiments, we can validate its robustness to such physical differences and its ability to capture the behavior of new phenotypes.

Three candidates exist for the cause of behavioral abnormalities seen in such mutants; differences in the worm's neural circuit, muscle properties, or passive body properties. We make clear experimental predictions on the locus of change and the specific pathways through which it is effected.

